# Modelling the impact of climate change on woody plant population dynamics in South African savanna

**DOI:** 10.1186/1472-6785-4-17

**Published:** 2004-12-17

**Authors:** Jörg Tews, Florian Jeltsch

**Affiliations:** 1 Plant Ecology and Nature Conservation, Institute of Biochemistry and Biology, University of Potsdam, Maulbeerallee 2, D-14467 Potsdam, Germany; 2 Plant Ecology and Nature Conservation, Institute of Biochemistry and Biology, University of Potsdam, Maulbeerallee 2, D-14467 Potsdam, Germany

## Abstract

**Background:**

In Southern Africa savannas climate change has been proposed to alter rainfall, the most important environmental driver for woody plants. Woody plants are a major component of savanna vegetation determining rangeland condition and biodiversity. In this study we use a spatially explicit, stochastic computer model to assess the impact of climate change on the population dynamics of *Grewia flava*, a common, fleshy-fruited shrub species in the southern Kalahari. Understanding the population dynamics of *Grewia flava *is a crucial task, because it is widely involved in the shrub/bush encroachment process, a major concern for rangeland management due to its adverse effect on livestock carrying capacity and biodiversity.

**Results:**

For our study we consider four climate change scenarios that have been proposed for the southern Kalahari for the coming decades: (1) an increase in annual precipitation by 30–40%, (2) a decrease by 5–15%, (3) an increase in variation of extreme rainfall years by 10–20%, (4) and increase in temporal auto-correlation, i.e. increasing length and variation of periodic rainfall oscillations related to El Niño/La Niña phenomena. We evaluate the slope *z *of the time-shrub density relationship to quantify the population trend. For each climate change scenario we then compared the departure of *z *from typical stable population dynamics under current climatic conditions. Based on the simulation experiments we observed a positive population trend for scenario (1) and a negative trend for scenario (2). In terms of the projected rates of precipitation change for scenario (3) and (4) population dynamics were found to be relatively stable. However, for a larger increase in inter-annual variation or in temporal auto-correlation of rainfall population trends were negative, because favorable rainfall years had a limited positive impact due to the limited shrub carrying capacity.

**Conclusions:**

We conclude that a possible increase in precipitation will strongly facilitate shrub encroachment threatening savanna rangeland conditions and regional biodiversity. Furthermore, the negative effects found for positive auto-correlated rainfall support current ecological theory stating that periodically fluctuating environments can reduce population viability because species suffer disproportionately from poor environmental conditions.

## Background

In order to assess biodiversity response under climate change, Hannah et al. [1] recently emphasized the need to apply simulation models operating on a regional scale. Moreover, species respond differently to climate change because of different adaptations to their environment [2]. As a consequence, single-species models with a regional focus are essential to fully understand the manifold impact of global climate change. However, even though recent simulation tools have occasionally been applied for climate-sensitive animal species (e.g. [3-7]), spatial plant population models are extremely scarce (see review in [8]).

In this study we show how climate change may affect the long-term population dynamics of the raisin bush, *Grewia flava *DC, a common, fleshy-fruited, woody plant species of South African savannas. Understanding the population dynamics of *Grewia flava *is a crucial task, because it is widely involved in the shrub/bush encroachment process (e.g. [9,10]). Shrub encroachment, i.e. the increase in woody plant cover, is a major concern for conservation and savanna rangeland management, due to its adverse effect on livestock carrying capacity (e.g. [11-13]) and biodiversity (e.g. [14]). In the context of global climate change, increase in woody plant cover has primarily been investigated in association with elevated CO_2 _(e.g. [15,16]). For example, Bond et al. [16] suggested that higher rates of atmospheric CO_2 _will have a positive effect on the post-fire regrowth of woody plants resulting in an increase in woody plant cover. Unfortunately, appropriate data verifying this hypothesis are not yet available.

Here, we present a different approach. We used *Spatial Grewia Model (SGM) *(see [17,18]), a stochastic, spatially-explicit computer model to evaluate the impact of precipitation pattern change on *Grewia flava *population dynamics. In semi-arid and arid savannas, rain is the most important environmental parameter governing crucial life history processes in woody plants [19]. Hence, climate change related shifts in precipitation pattern will potentially have severe consequences for woody plant population dynamics. As a regional focus for our study we considered the southern Kalahari, the near centre of *Grewia flava*'s distribution. In this area, recent climatological studies proposed either a decrease in mean precipitation of 5–15% by the year 2050 [20], or an increase by up to 30–40% (e.g. [21]. Further studies suggest an increase in the frequency and variability of extreme rainfall events (e.g. [22]), as well as alternating phases with low and high rainfall, typical for Southern Africa [23]. The large divergence between the various precipitation scenarios raises the question how woody plants would react along this spectrum. Therefore, we have set up a systematic sensitivity study to explore *Grewia flava *population dynamics along precipitation gradients, so as to detect possible thresholds in the response of this species to continuous variations of a crucial environmental driver.

## Results

**Figure 1:**
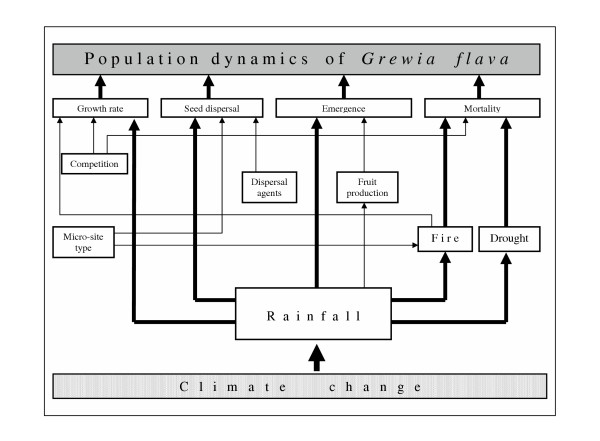
Flow chart of *SGM *showing causal pathways of *Grewia flava *population dynamics. Bold arrows indicate processes where population parameters and variables are affected by the annual type of rainfall (for detailed model description see text).

**Figure 2:**
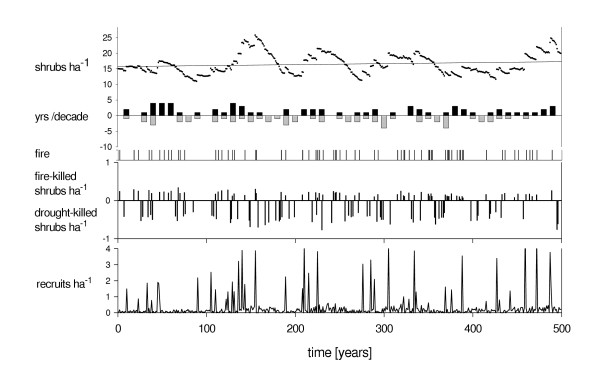
Typical *SGM *time series of the standard scenario with 500 annual time steps. Population trend is given as shrub density with simple linear regression y = 0.0032x + 15.73. Mean slope of population trend for 100 simulation runs was *z *= 0.000049x + 13.41 with standard deviation of +/-0.017. Further model output from top to bottom: number of high (black) and low rainfall (grey) years per decade, fire occurrence (indicated as a black bar), density of fire- and drought-killed shrubs and density of recruits. Decades with frequent high rainfall years are indicated by frequent fires and increased fire mortality. Decades with exceptionally low rainfall show low fire frequency and increased drought mortality. Peaks of recruitment occur mostly in years with high rainfall and absence of fire.

By implementing relevant ecological processes of *Grewia flava *population dynamics into the *SGM *(see Figure 1 and model description in method section), we developed a standard scenario, based on annual rainfall for the period 1940 – 2000. This standard scenario led to stable population dynamics (see Figure 2 and method section). To compare population trends between four climate change scenarios we then used simple linear regression to calculate the *z*-value, i.e. the slope of the year – shrub density relationship.

### (1) Increase in precipitation

**Figure 3:**
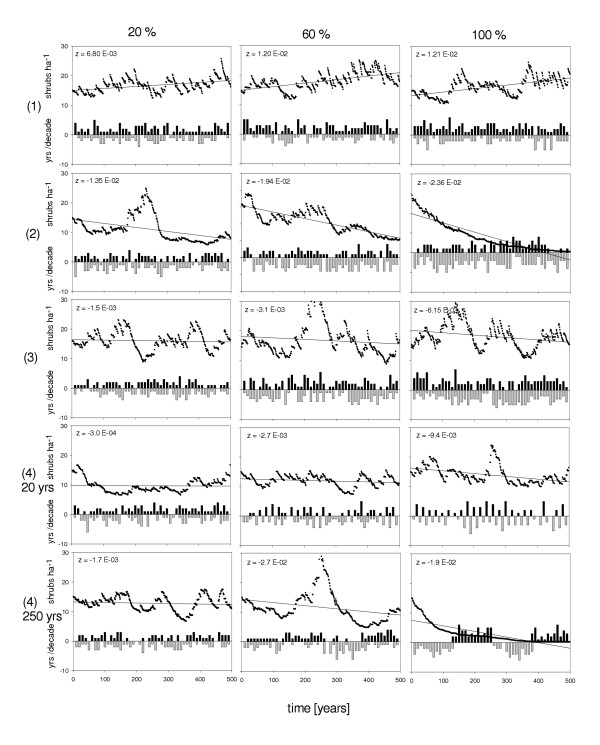
Typical time series for climate change scenarios with *z*-value of the population trend: increase in precipitation (1); decrease in precipitation (2); increase in inter-annual variation of precipitation (3); increase in temporal auto-correlation of precipitation (4) for period length *PL *with 20 and 250 years. Columns indicate 20%, 60% and 100% variation, respectively (see parameter values in Table 1).

**Figure 4:**
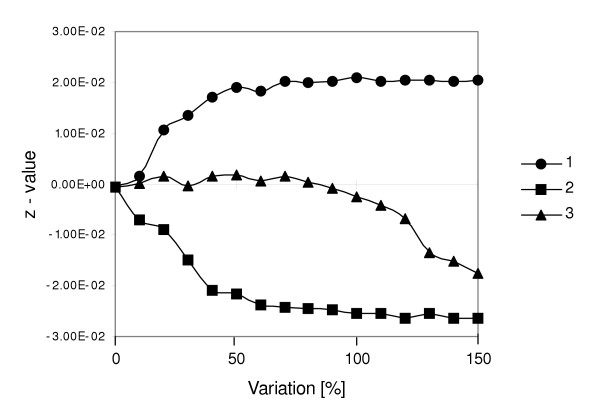
Results of simulation experiments for climate change scenarios according to: increase in precipitation (1), decrease in precipitation (2) and increase in inter-annual variation of precipitation (3) given as mean population trend *z *for 100 simulation replicates. The mean population trend is given as *z *for 100 simulation replicates plotted against percent variation in the probability of occurrence of extreme rainfall years as compared to the standard scenario.

An increase in the frequency of high rainfall years resulted in an increase of the *z*-value (Figure 3 and Figure 4). For example, a 20% increase in high rainfall years yielded a mean *z*-value of 0.01 (Figure 4). With an increase of 50%, the maximum *z*-value was 0.02. This upper limit could be clearly assigned, because the carrying capacity of cell type *T *had been reached, i.e. no further recruits were able to establish in the sub-canopy of trees. Even though additional juveniles may still emerge in cell type *M*, they are prevented from reaching the open due to a low probability of *P*_*matrix*_. Additionally, fire mortality is higher in cell type *M*.

### (2) Decrease in precipitation

A decrease in mean precipitation resulted in a negative population trend (scenario 2, Figure 3) with a lower limit of *z *at -0.027 (Figure 4). A 10% increase of P_*low*_, corresponding to a probability of occurrence of low rainfall years of P_*low *_= 0.165, yielded a mean *z *value of -0.008. This continued to *z *= -0.02 for a 40% increase of P_*low*_. Above a 60% increase of P_*low *_30% of all replicate runs resulted an extinction of the *Grewia flava *population within the 500 year time frame (not shown, however compare Figure 3). Comparing the upper and lower limits of the *z-*value for scenario (1) and (2), negative effects associated with an increase in drought events were more effective than the positive effects related to higher recruitment rates associated with comparable rainfall excess (0.02 as opposed to -0.027 see Figure 4).

### (3) Increase in inter-annual variation of precipitation

An increase in inter-annual variation between low and high rainfall years below 100% did not cause any significant departure from stable population dynamics. However, above a threshold value of 100%, corresponding to a reduction of P_*average *_from 0.72 to less than 0.44 (see Table 1), we observed a decrease in the mean *z*-value. For example, for an increase in variation of 150%, *z *was -0.02 (Figure 4). Here, rainfall almost exclusively occurred as either high or low (P_*low *_= 0.375, P_*high *_= 0.325). Considering the negative and positive effects of low and high rainfall years, negative effects, such as drought, outweighed positive effects associated with enforced recruitment in high rainfall years.

**Table 1:** Probability values for low (first value) and high rainfall years (second value) for climate change scenarios: increase in precipitation (1); decrease in precipitation (2); increase in inter-annual variation of precipitation (3); increase in temporal auto-correlation of precipitation (4) with 'good' phase (4_a_) and 'poor' phase (4_b_). Columns indicate 20%, 60% and 100% variation in the probability of occurrence of extreme rainfall years, respectively, as compared to the standard scenario (P_*low *_= 0.15, P_*average *_= 0.72, P_*high *_= 0.13).

Scenarios / Variation		20 %	60 %	100 %
(1)	P_*low*_	0.150	0.150	0.150
	P_*high*_	0.156	0.208	0.260
(2)	P_*low*_	0.180	0.240	0.300
	P_*high*_	0.130	0.130	0.130
(3)	P_*low*_	0.180	0.240	0.300
	P_*high*_	0.156	0.208	0.260
(4_a_) 'good' phase	P_*low*_	0.120	0.060	0.000
	P_*high*_	0.156	0.208	0.260
(4_b_) 'poor' phase	P_*low*_	0.180	0.240	0.300
	P_*high*_	0.104	0.052	0.000

### (4) Increase in temporal auto-correlation of precipitation

**Figure 5:**
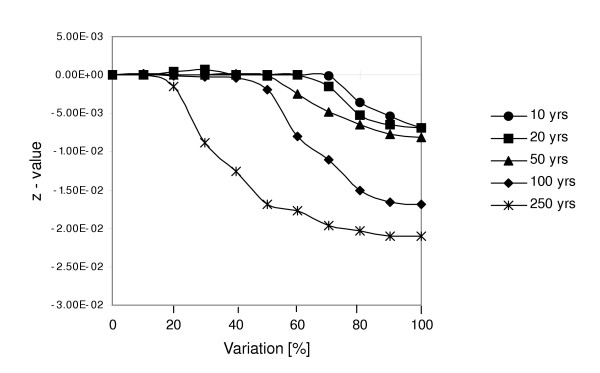
Results of simulation experiments for increase in temporal auto-correlation of precipitation (climate change scenario 4) according to a *PL *value of 10, 20, 50, 100 and 250 years. The mean population trend is given as *z *for 100 simulation replicates plotted against percent variation in the probability of occurrence of extreme rainfall years as compared to the standard scenario.

Despite constant mean and inter-annual variation in precipitation, increasing period length (*PL*) and intra-cycle variation (*ICV*) led to negative population trends and a decrease in *z*-value (Figure 5). Here, we found varying *ICV *thresholds for the *PL *scenarios tested, i.e. an abrupt departure from stable population dynamics. For example, a 10-year alternating rainfall cycle with an increase of 80 % in *ICV *(corresponding to a 5-year 'poor' phase with P_*low *_= 0.27 / P_*high *_= 0.026 and a 'good' phase with P_*low *_= 0.03 / P_*high *_= 0.234) yielded a *z*-value of -0.003, whereas population dynamics were stable for an *ICV *of 70%. Further thresholds were found above 60% *ICV *for a *PL *value of 20 years, 50% for 50 years, 40% for 100 years and 15% for 250 years. In the latter case, a large proportion of the population became extinct below 50% *ICV *where *z *was close to its lower limit. It is noteworthy, that we found no significant difference between similar *PL *scenarios that began with different phase conditions.

## Discussion

Based on our simulation results, we have demonstrated that climate change predictions of precipitation pattern for the southern Kalahari may significantly affect *Grewia flava *population dynamics. This enables us to estimate the possible consequences for land use management and biodiversity due to the keystone function of *Grewia flava *in the shrub/bush encroachment process.

### What are the implications of climate change related population trends for regional biodiversity and rangeland condition?

#### (1) Increase in precipitation

A climate change related increase in precipitation is commonly rejected [20]). However, based on a 28-year atmospheric circulation model, Mason et al. [21] proposed an increase in mean annual rainfall of 30–40% for the southern Kalahari. Yet, due to the model's insensitivity to topography, the authors also emphasize uncertainties in their predictions. This scenario would result in a strong increase in shrub density as indicated by a positive mean population trend for the *SGM *simulation period (Figure 4). Here, fire and drought mortality rates are too low to compensate substantial increase in juvenile recruitment associated with high rainfall years. On rangelands with domestic livestock this process may be strongly enhanced: in an earlier model version it was shown that cattle feeding on the foliage of *Grewia flava *may disperse seeds into the open matrix vegetation through dung deposition and thus facilitate shrub encroachment [18]. In this case we propose that an increase in precipitation will result in a catastrophic deterioration of current rangeland conditions within several decades. As a further consequence, biodiversity will most likely decrease due to the homogenization of woody plant community structure [24]. An increase in structural homogeneity due to shrub encroachment has been shown to adversely affect species diversity in both plants and animals (e.g. [14,25]).

#### (2) Decrease in precipitation

The majority of climatological studies propose a decrease in annual precipitation of 5–15% by the year 2050 [20]. For a 10% increase in the probability of occurrence of low rainfall years (at the cost of average years) we found a negative trend for *Grewia flava *population dynamics (Figure 3). High frequency of low rainfall years resulted in an increase of drought-related mortality and the reduction of shrub density. Increases in the probability of occurrence of low rainfall years of more than 40% lead occasionally to population extinction within the *SGM *time frame. However, we believe that under natural conditions population dynamics of *Grewia flava *may also stabilize at a lower level, as suggested by its distribution in arid parts of the southern Kalahari where droughts are more frequent (e.g. [26]). Even though precipitation decrease may mitigate the risk of local *Grewia *encroachment, a strong reduction in shrub density may lead to cascade effects in the food chain because the fruits are an important component in the diet of animals and humans in an environment where food resources are otherwise scarce [27].

#### (3) Increase in inter-annual variation of precipitation

The inter-annual variation in precipitation, i.e. frequency and magnitude of extreme rainfall events, has been predicted to increase with ongoing climate change within the next decades (e.g. [22,28]). Rates may range between 10–20% for the southern Kalahari [21]. For this scenario *SGM *produced no significant departure form stable population dynamics (Figure 4). Thus, we propose that the predicted increase in inter-annual variability will have a low impact on the natural population dynamics and shrub density of *Grewia flava*. However, this may differ for rangelands with high cattle grazing: an earlier model version showed that increase in the probability of extreme rainfall years can increase shrub encroachment if additional seed dispersal by cattle into the matrix is considered [18].

An increase in variability of more than 100% revealed a significant threshold behavior with a negative *Grewia flava *population trend. Above this threshold frequent droughts kill the offspring and inhibit emergence. Ecological theory generally suggest that population viability decreases with increase in environmental variability or stochasticity [29], because favorable events have a limited positive impact due to the carrying capacity, whereas unfavorable events have a full negative impact [7]. However, to our knowledge, the general character of this relationship has not been studied in detail yet. Thus, the pattern described here provides new insights into the relationship between environmental variability and the corresponding population dynamics.

#### (4) Increase in temporal auto-correlation of precipitation

Tyson [23] showed evidence of periodic, non-random rainfall oscillations with a period of ca. 18–20 years in Southern Africa. This distinct pattern has recently been associated with El Niño/La Niña phenomena and may increase under climate change [30]. Since we did not simulate actual total rainfall we used values of *ICV*, i.e. intra-cycle variation and *PL*, i.e. period length to evaluate the impact of climate change related increase in positive auto-correlation of rainfall. In our study region, Kruger [30] found an oscillation with a *PL *value of 22 years between 1955 and 1991. The *ICV *values used in our simulation experiments refer to an increase in the probability of an extreme rainfall category and are thus difficult to compare with the cyclic variability of actual total rainfall. However, we compared the *ICV *values from our model with departures of annual rainfall from the inter-annual mean of a typical rainfall oscillation provided by Kruger [30]. Based on this, we infer that an *ICV *value above 60% resembles a realistic rainfall variation between favorable and unfavorable periods of a 22-year oscillation. Although we did not find a negative impact on *Grewia flava *population dynamics for this scenario, a slight increase in *ICV *combined with an increasing length of El Niño/La Niña phases might have a significant negative impact on the population viability of *Grewia flava *(see Figure 5). This will have profound consequences for other organisms which depend on, for example, fruit provision.

As a further corollary, our results confirm current ecological theory that positive auto-correlation with constant average and inter-annual variation of a driving environmental parameter can reduce population viability and lead to extinction (e.g. [17,31,32]). In terms of the internal model processes of *SGM*, this means that negative effects in drought years (i.e. drought mortality and lack of reproduction) outweigh positive effects in wet years (i.e. increased reproduction). This is noteworthy, as mean annual rainfall and inter-annual variation was kept constant. Until now, this has been primarily shown in solely theoretical studies with under-compensating population dynamics (e.g. [33]) or individual-based animal models (e.g. [7]). To our knowledge this is the first empirical-based, plant population model that showed evidence of population persistence being negatively influenced by positive auto-correlation through a periodically fluctuating environmental driver.

### Parameter sensitivity and predictive power of *SGM*

In order to assess the predictive power of *SGM *it is important to discuss the inference of actual precipitation amounts on probability values. Threshold values for extreme rainfall years are based on expert knowledge and may vary depending on the plant species considered. Moreover, percentage changes in extreme rainfall years, as applied in our model, are not necessarily equivalent to percentages changes in mean annual rainfall. For example, an increase in 'high' rainfall years by 10% represents an increase in mean annual rainfall of approximately 10%, depending on actual precipitation amounts in each 'high' rainfall year. Thus, it is important to derive principal population trends from the *SGM *model results rather than absolute predictions. Another issue regards the temporal dimension: long-term *SGM *simulation periods deviate from short-term predictions of climate change. However, population trends applied for only a few decades may yield biased results due to the cyclicity of *Grewia *population dynamics (see [17]). Finally, we stress that potential deviations in the annual probability of drought mortality may modify the output of the model. For example, a sensitivity analysis showed that a 100% increase in drought mortality for adult shrubs decreased the model output by 10% (see [18]).

## Conclusions

In this study we have shown that climate change may have severe and sometimes unexpected implications at a regional species level. A woody plant like *Grewia flava *is relatively long-lived, fire and drought-resistant. Intuitively, this would suggest a low climate change impact on *Grewia flava *population dynamics. However, we found that despite *Grewia flava*'s capability to survive in a harsh environment, it may be strongly affected when rainfall decreases as predicted, or increases in periodical fluctuations. This may also include possible range shifts in regional distribution which have not been studied here. Based on the model and the climate change scenarios analysed, it would be inappropiate to forecasts changes in the geographical distribution of *Grewia flava*, because *SGM *parameters have been estimated and validated for populations in the Kimberley region of the southern Kalahari. Moreover, current geographical distribution suggests that buffer mechanisms may facilitate survival in more arid parts of the southern Kalahari (e.g. through higher variability in emergence as an adaption towards lower frequency of high rainfall years).

Decrease in rainfall may reduce the severity of *Grewia flava *encroachment in the southern Kalahari. However, increase in rainfall will likely enhance this process. A shift from typical open grassland with solitary trees towards wide spread, homogeneous *Grewia flava *thickets implies negative cascade effects for other species, resulting in the loss of biodiversity [24]. As a further consequence, local rangelands are likely to be reduced in their carrying capacity of domestic livestock with negative effects for economic sustainability. Moreover, a shift in precipitation pattern may have further consequences, such as the alteration of fire regimes, grass biomass production or woody plant carbon uptake.

## Methods

A detailed description of the simulation model *SGM *and previous results have been presented elsewhere (see [17,18]). *SGM *has been previously validated with empirical data and simulates population dynamics of *Grewia flava *under specific land use, fire and rain scenarios in southern Kalahari semiarid savannas. However, to facilitate a better understanding of the current results we will briefly describe the study species and relevant aspects of the simulation model.

### The study species

In the open savannas of the southern Kalahari, *Grewia flava *typically grows beneath the dominant tree species *Acacia erioloba *[34], because bird-mediated seed dispersal predominately confines new establishments to woody plant microsites. However, occasionally large individuals may be found in the open grassland matrix at former tree sites, suggesting high longevity of *Grewia flava*. Under high cattle grazing a substantial proportion of seeds may be distributed into the open matrix vegetation, since cattle feed on the foliage and fleshy fruits of *Grewia flava *[18]. This may result in a substantial increase in *Grewia *cover, particularly around boreholes (e.g. [10]). *Grewia flava *has excellent resprouting capabilities after fire [35] and low drought mortality rates [36]. Size class distributions suggest a demographic bottleneck in early life stages due to low rates of emergence and high juvenile mortality [36].

### General model structure

The computer model *SGM *(see [17,18]) represents a grid-based approach iteratively simulating population dynamics of *Grewia flava *in annual time steps for a period of 500 years. Based on empirical demographic and spatial data from the Kimberley region of the southern Kalahari (see [36]), an initial population of 15 shrubs ha^-1 ^was distributed on a 200 × 200 cell grid, with each cell representing 5 m × 5 m of savanna vegetation. The *SGM *grid is developed in two layers: a landscape and a population layer. In the first layer micro-site types of the savanna vegetation may change in the course of time. In the second layer *SGM *simulates population dynamics of *Grewia flava*. A cell type was classified as either tree (*T*) (i.e. occupied by *Acacia erioloba*) or matrix type (*M*) (i.e. grassland vegetation). However, it switched from *T *to *M *status when a tree died after a mean life span of 200 years. The converse occurred where a new tree establishes. Initial tree distribution and spatial recruitment pattern was random with a constant density of 5 trees ha^-1 ^for the entire simulation period. In each time step, *SGM *simulated important life history stages, environmental conditions and the key ecological processes of *Grewia flava*, the majority of which are governed by annual rainfall patterns (see Figure 1). For example in the model, rainfall directly determines the likelihood of a fire, drought and fire mortality, fruit crop size, emergence and annual shrub growth rate.

### Rainfall

Annual rainfall in the southern Kalahari is highly variable, with precipitation ranging between 200 and 700 mm yr^-1^. Rain falls almost exclusively during summer (November to April) with an inter-annual mean of 417 mm for the period 1940 – 2000 (Kimberley airport, South African Weather Service 2001, unpublished data). For this period we defined a threshold value of 150 mm below and above the long-term mean to classify years into 'low', 'average' and 'high' rainfall years (thresholds of 267 mm yr^-1^and 567 mm yr^-1^, respectively) (see [18]). Accordingly, frequency of extreme rainfall years resulted in an annual probability of P_*low *_= 0.15 for low and P_*high *_= 0.13 for high rainfall years (P_*average *_= 0.72). Similar classifications have been applied in other studies, e.g. in a spatial simulation model of *Acacia raddiana *in the Negev Desert [37].

### Fruit production and seed dispersal

Fruit production rates are based on mean crop size of *Grewia *size classes and may vary depending on the annual rainfall (see [18]). Seed dispersal is mostly zoochorous and spatially aggregated with a large proportion of seeds deposited in woody plant microsites. It is a crucial factor for the population dynamics and long-term viability of *Grewia flava *[36] and represented by two parameters: probability of seeds removed from a shrub and deposited in cell type *M*, *P*_*matrix*_, and cell type *T*, *P*_*acacia*_. *P*_*matrix *_varies randomly per year between 0.1–0.01% and represents occasional seed distribution through, e.g. small mammals. *P*_*acacia *_refers to bird-mediated seed dispersal with a range of 1–5% in high rainfall years, 2.5–7.5% in average and 5–10% in low rainfall years. Based on estimates from empirical data [36], the assumption of relatively constant seed dispersal rates may be reasonable as the proportion of seeds removed is most likely to be higher in years of lower fruit set. Fruit production typically varies more than bird abundances, and unless birds switch in exactly compensatory fashion to other fruits to the degree that *Grewia flava *becomes less abundant, fruit removal may be higher in years of low population size.

### Emergence

Even though microsite types differ markedly in micro-environmental conditions, they do not differ in emergence rates of *Grewia flava *seeds [36] suggesting a similar emergence probability for cell type *M *and *T*. However, depending on annual rainfall conditions, the probability of emergence in the model may vary between 1–2% in average and 2–4% in high rainfall years (no emergence occurs in low rainfall years). As survival of *Grewia flava *seeds in the soil is very low, seeds that do not emerge are assumed to die.

### Fire and drought

The occurrence and intensity of a fire depends on the amount of rainfall, since precipitation determines annual grass biomass production and thus fuel load [38]. In the *SGM*, fire was simulated probabilistically for the entire grid and may only occur in high and average rainfall years. Average frequency of 7.9 years in the model is supported by fire intervals reported from similar savannah types [39]. The impact of fire varies spatially and demographically: a seedling in the matrix vegetation has a 95% chance of being killed in a fire, compared with 4% for adult shrubs which largely resprout in the following year. For *T *cells, fire mortality probability is 0% for adults and 75% for seedlings. These model assumptions are realistic, since grass fuel accumulation and fire severity beneath trees is less than in tree inter-spaces (e.g. [40,41]). In the *SGM*, the probability of drought mortality varies between life stages and the annual rainfall conditions (see also model description in [18]). Annual drought mortality for adults is restricted to low rainfall years with a probability of 3% for both cell types. This assumption is based on data from O'Connor [42] and Schurr [36]. For seedlings the annual drought mortality probability is 90% for average and 50% for high rainfall years (see [18]). All seedlings are assumed to die in years with low rainfall conditions.

### Growth

In the model, each shrub was assigned a size class with an average canopy cover. For definition of shrub size classes we used the canopy volume to group *Grewia flava *individuals into categories of small (*Grewia*_*S*_; <1 m^3^), medium-sized (*Grewia*_*M*_; 1–10 m^3^) and large plants (*Grewia*_*L*_; >10 m^3^) as well as seedlings (*Grewia*_*seedling*_; temporary state after emergence, transforming to *Grewia*_*S *_with the following year). We assumed a maximum age for each size class member with *MaxAge*_*S *_= 5 years for small and *MaxAge*_*M *_= 25 years for medium-sized shrubs. These estimates are based on annual shoot growth rates and aerial photograph analysis of the study area (see [18]). In each annual time step an individual can accumulate a growth year and, if *MaxAge *is reached, attain the next size class with *P*_*transition*(*S*) _= 0.2 for small and *P*_*transition*(*M*) _= 0.1 for medium-sized shrubs. No growth occurs in low rainfall years.

### Carrying capacity and competition

To incorporate intra-specific competition we used a simple causal approach that incorporates cell-based shrub cover. Therefore, we defined a carrying capacity of *K *= 25 m^2 ^as maximum total cell shrub cover. If shrub cover exceeded *K*, individuals in the cell died. Density-dependent mortality was simulated annually by removing the smallest individuals first, i.e. in descending order of size class, until shrub cover <*K *(for further details see [18]). Inter-specific competition between *Grewia flava *seedlings and the grass layer were neglected: empirical tests showed that emergence rates were similar within and outside of the grassy vegetation matrix (see [36]). For the adult stage, inter-specific competition with other woody plants is of low importance because *Grewia flava *often occurs as a mono-dominant species, particularly on bush encroached rangelands.

### Simulation experiments

The standard scenario of the model, based on rainfall for the period 1940 – 2000, led to stable population dynamics (Figure 2). As standard deviation was generally high we performed 100 replicate simulation runs (± 0.0173 for the standard scenario, see Figure 2). To determine and compare population trends between the climate change scenarios we then used a simple linear regression of years vs. shrub density to calculate the *z*-value, i.e. the slope of the year – shrub density relationship. This is a reasonable approach for analyzing time-abundance relationships (e.g. [43]). When population dynamics were stable for 500 years, i.e. *z *was ≅ 0, the number of new recruits more or less resembled the number of fire- and drought-killed shrubs (Figure 2). Significant recruitment events mostly occurred in high rainfall years without fire.

Based on the standard scenario we performed simulation experiments for each of the four different scenarios of climate change with variation in inter-annual mean of precipitation, coefficient of inter-annual variation, and temporal auto-correlation (see Table 1):

(1) increase in precipitation, i.e. stepwise increase of P_*high *_resulting in a higher probability of high rainfall years (increase in recruitment events). Probability of low rainfall years is constant whereas frequency of average years is reduced, accordingly.

(2) decrease in precipitation, i.e. stepwise increase of P_*low *_resulting in a higher probability of low rainfall years (increase in drought events),

(3) increase in inter-annual variation of precipitation, i.e. stepwise increase of P_*low *_and P_*high *_resulting in a higher probability of low and high rainfall years at the cost of average years (unaltered inter-annual mean),

(4) increase in temporal auto-correlation of rainfall with 'good' and 'poor' phases.

For the scenario (4) we varied two parameters:

(4a) introduction of rain cycles with increasing period length *PL*, i.e. longer phases of favorable and unfavorable rain conditions and,

(4b) increase in intra-cycle variation *ICV*, i.e. increasing variation between alternating favorable and unfavorable periods within one rain cycle.

For *PL *we applied a period length of 10, 20, 50, 100 and 250 years, respectively, with each period subdivided into a similarly long 'good' and 'poor' phase. For example, a 10-year period length was subdivided into a 5-year period of superior and a 5-year period of poor conditions. For an increase in *ICV *we alternately increased and decreased high and low rainfall probabilities in each period, respectively (see 4_a _and 4_b _in Table 1). For example, for an *ICV *value of 100% P_*low *_equaled 0.00 and P_*high *_0.26 in a 'good' phase, whereas values were 0.30 and 0.00, respectively for a 'poor' phase. Through this procedure inter-annual mean and variation of rainfall probabilities were identical for each scenario and the default set of rainfall probabilities. Combined increase in *PL *and *ICV *resulted in an increase of positive auto-correlation. Near-decadal epochs of above- and below-normal rainfall have been identified for the period 1955–1991 [30] and may increase in *ICV *under predicted climate change.

## Author's contribution

JT developed the *SGM *simulation model, conceived the study and drafted the manuscript. FJ supervised the study and the manuscript writing. Both authors read and approved the final manuscript.
